# Rabies-Related Knowledge and Practices Among Persons At Risk of Bat Exposures in Thailand

**DOI:** 10.1371/journal.pntd.0001054

**Published:** 2011-06-28

**Authors:** Kis Robertson, Boonlert Lumlertdacha, Richard Franka, Brett Petersen, Saithip Bhengsri, Sununta Henchaichon, Leonard F. Peruski, Henry C. Baggett, Susan A. Maloney, Charles E. Rupprecht

**Affiliations:** 1 Epidemic Intelligence Service, Office of Workforce and Career Development, Centers for Disease Control and Prevention (CDC), Atlanta, Georgia, United States of America; 2 Queen Saovabha Memorial Institute (WHO Collaborating Centre for Research on Rabies), Thai Red Cross Society, Bangkok, Thailand; 3 Poxvirus and Rabies Branch, Division of High-Consequence Pathogens and Pathology, Centers for Disease Control and Prevention (CDC), Atlanta, Georgia, United States of America; 4 International Emerging Infections Program, Global Disease Detection and Emergency Response Division, Centers for Disease Control and Prevention (CDC), Bangkok, Thailand; University of Oklahoma Health Sciences Center, United States of America

## Abstract

**Background:**

Rabies is a fatal encephalitis caused by lyssaviruses. Evidence of lyssavirus circulation has recently emerged in Southeast Asian bats. A cross-sectional study was conducted in Thailand to assess rabies-related knowledge and practices among persons regularly exposed to bats and bat habitats. The objectives were to identify deficiencies in rabies awareness, describe the occurrence of bat exposures, and explore factors associated with transdermal bat exposures.

**Methods:**

A survey was administered to a convenience sample of adult guano miners, bat hunters, game wardens, and residents/personnel at Buddhist temples where mass bat roosting occurs. The questionnaire elicited information on demographics, experience with bat exposures, and rabies knowledge. Participants were also asked to describe actions they would take in response to a bat bite as well as actions for a bite from a potentially rabid animal. Bivariate analysis was used to compare responses between groups and multivariable logistic regression was used to explore factors independently associated with being bitten or scratched by a bat.

**Findings:**

Of 106 people interviewed, 11 (10%) identified bats as a potential source of rabies. A history of a bat bite or scratch was reported by 29 (27%), and 38 (36%) stated either that they would do nothing or that they did not know what they would do in response to a bat bite. Guano miners were less likely than other groups to indicate animal bites as a mechanism of rabies transmission (68% vs. 90%, p = 0.03) and were less likely to say they would respond appropriately to a bat bite or scratch (61% vs. 27%, p = 0.003). Guano mining, bat hunting, and being in a bat cave or roost area more than 5 times a year were associated with history of a bat bite or scratch.

**Conclusions:**

These findings indicate the need for educational outreach to raise awareness of bat rabies, promote exposure prevention, and ensure appropriate health-seeking behaviors for bat-inflicted wounds, particularly among at-risk groups in Thailand.

## Author Summary

Rabies is a fatal encephalitis caused by lyssaviruses. Evidence of lyssavirus circulation has recently emerged in Southeast Asian bats. We surveyed persons regularly exposed to bats and bat habitats in Thailand to assess rabies‐related knowledge and practices. Targeted groups included guano miners, bat hunters, game wardens, and residents/personnel at Buddhist temples where mass bat roosting occurs. Of the 106 people interviewed, 11 (10%) identified bats as a source of rabies. History of a bat bite/scratch was reported by 29 (27%), and 38 (36%) expressed either that they would do nothing or that they did not know what they would do in response to a bat bite. Guano miners were less likely than other groups to indicate animal bites as a mechanism of transmission (68% vs. 90%, p=0.03) and were less likely to say they would respond appropriately to a bat bite or scratch (61% vs. 27%, p=0.003). These findings indicate a need for educational outreach in Thailand to raise awareness of bat rabies, promote exposure prevention, and ensure health‐seeking behaviors for bat‐inflicted wounds, particularly among at‐risk groups.

## Introduction

Rabies is an exceptionally fatal encephalitis caused by Rhabdoviruses in the Lyssavirus genus. Transmission typically occurs when broken skin is contaminated with saliva from an infected mammal—usually in association with a bite but in rare instances by scratches. The most well-known and ubiquitous lyssavirus is the rabies virus (RABV), which circulates in New World bats and both Old and New World terrestrial mammals. The vast majority of human rabies cases worldwide are transmitted by dogs infected with RABV. A lesser known member of the genus is the Mokola virus, which has been isolated from a number of terrestrial mammals in Africa (most notably shrews) and has caused at least two human cases [1],[2]. Reservoirs for the remaining nine members of the Lyssavirus genus appear to be exclusively Old World bats [3].

Rabies is a major public health problem in Asia. Of the estimated 55,000 human cases that occur annually worldwide, more than half occur in Asian countries [4]. In recent decades, initiatives aimed at raising rabies awareness (e.g. the World Rabies Day campaign) and lowering human exposure risk through mass vaccination of leading reservoir species have been implemented globally, coinciding with the development of highly potent human rabies vaccines [4],[5],[6]. Notable trends have subsequently followed. Of all Asian countries, Thailand has experienced the steadiest decline in human rabies cases, with a near 10-fold decrease in reported cases during the last 20 years [7]. Much of this decline is attributable to the country's very extensive use of rabies vaccine in the treatment of persons bitten by dogs. In 2003, for instance, more than 400,000 people in Thailand were vaccinated against rabies following potential rabies exposures [8].

Historically in Southeast (SE) Asia, animal-based prevention efforts for rabies have almost exclusively been centered on dogs. Most reported human cases in the region are traced to these animals either through an exposure history or through molecular or antigenic subtyping of variants from rabid human patients [9],[10]. The canine-associated rabies variant has been the only one linked to terrestrial wildlife and domestic animals in Thailand [8], further evidence that dogs are the main lyssavirus reservoir in the region. To date, no human cases of rabies linked to lyssaviruses other than canine-associated RABV have been reported in Thailand or the rest of SE Asia. Like other dog-rabies endemic countries, the majority of human rabies victims in Thailand are children under the age of 15 years [11].

Within the last ten years, however, interest in bats and their role in lyssavirus transmission has increased in the region. The discovery of the Australian Bat Lyssavirus (ABLV) in Australian flying fox bats (*Pteropus spp.*) in the mid-1990's and the isolation of new bat lyssaviruses in the former Soviet Union [12],[13] were pivotal in turning scientific interest towards the study of potential Asian bat reservoirs. In the last 10 years, evidence of lyssavirus maintenance in SE Asian Chiropterans has emerged from surveillance in Cambodia, Thailand, Bangladesh, and the Philippines [14],[15],[16],[17]. Although lyssaviruses have not yet been isolated from these mammals, neutralizing antibodies associated with lyssaviruses have been detected in sera from both regional mega and microbats. These findings strongly suggest that Asian bats maintain lyssaviruses like their counterparts in Europe, Africa, Australia, and the Americas. Human deaths due to bat-borne rabies infection have been well documented in these continents [12], [18], [19], [20]. In particular, rabid vampire bats are a major cause of human mortality in South America's Amazon region [21].

Routine surveillance for bat rabies is lacking in Asia and as a consequence, understanding is limited regarding the extent of lyssavirus circulation among SE Asian bats and the impact on animal and public health. However, evidence thus far raises pressing questions about human health risks. The potential implications of bat rabies are particularly salient in SE Asia because human-bat interaction occurs routinely in many locales. Bat guano is regularly mined from caves for use as a fertilizer. Hunting of bats for sale and personal consumption occurs as well, despite laws to stop this practice. The presence of large numbers of bats at many Buddhist temples also promotes exposures, as these sites are focal points for commerce, tourism, and religious expression.

Because the severity of skin trauma inflicted by bats is usually minor and unlikely to prompt a medical visit on the basis of physical injury alone, public knowledge of appropriate health-seeking behaviors following a bat exposure is especially important in preventing cases of bat-borne rabies. Rabies postexposure prophylaxis (PEP) for bat bites and scratches, as recommended by both the World Health Organization (WHO) and the U.S. Advisory Committee on Immunization Practices (ACIP), includes thorough wound washing and the administration of rabies immune globulin (in non-immunized individuals) and rabies vaccine administered in a series of doses [4],[22]. When administered promptly and properly, rabies PEP is highly effective in preventing the disease. To date, however, no intervention has proven effectiveness in stopping the clinical course after symptom onset, a fact which further underscores the importance of early care following a possible lyssavirus exposure.

Little is known about the extent of bat-specific rabies awareness in SE Asia. To assess the knowledge and practices of individuals who are most at-risk of bat exposures in Thailand, we surveyed persons who regularly come in contact with bats or bat dwellings through occupational activities and other practices. We sought to elucidate gaps in knowledge that potentially have bearing on rabies prevention, describe the occurrence of bat-associated exposures in this population, and explore factors associated with bat exposures that are of potential consequence to lyssavirus transmission.

## Methods

### Study Design and Population

**Figure 1:**
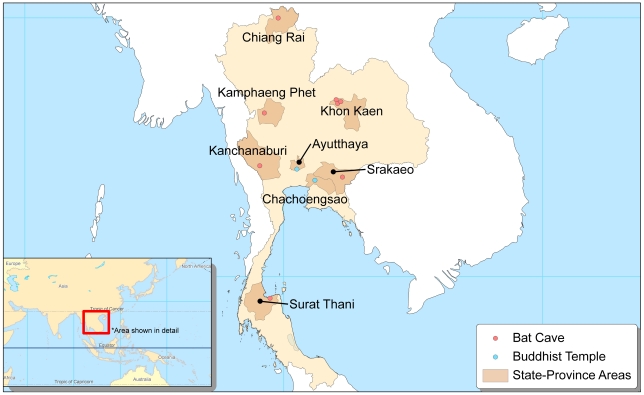
Recruitment areas for survey participation in Thailand.

The desired sample size was 200 individuals. Surveyed individuals were a convenience sample of adults who collect guano from caves, engage in bat hunting, work or reside at temples that serve as sites for mass bat roosting, or work as game wardens responsible for monitoring and protecting bat caves. Engagement in at least one of these activities within the last 5 years and being 18 years or older were inclusion criteria for participation. Individuals were recruited from eight provinces as shown by Figure 1. Recruitment areas were selected based on proximity to bat caves and/or mass bat roosting sites and certain community characteristics known to promote bat-associated activities (e.g., an agrarian-economy that benefits from guano fertilizer use). Participants were classified based on the activity in which they most frequently engaged, and were primarily recruited from rural and semi-rural localities. In farming communities near bat caves, village leaders and other local contacts provided assistance in locating individuals known to engage in guano mining. Such individuals were also found through referral from existing participants. A similar method was employed to locate bat hunters/trappers in communities where fruit farms are known to attract flying fox bats. To recruit temple workers/residents and game wardens, permission from supervisory officials at Buddhist temples and national parks was obtained before individuals under their management were approached for participation. Recruits were not offered or given incentives for participating.

### Ethics Statement

The study design and consent process was approved by the Institutional Review Board (IRB) at CDC (protocol# 5709). All participants were verbally informed of the study's purpose and assured that their responses would be kept anonymous, even if they engaged in illegal activities. Oral consent was obtained to ensure anonymity and accommodate illiterate participants, and was documented by the interviewer electronically via personal digital assistants (PDA) prior to administering the survey. This method of obtaining informed consent was approved by CDC's IRB.

### Questionnaire

A 41-item structured questionnaire was developed in English and translated and reviewed by native Thai speakers employed by the office of the U.S. CDC, International Emerging Infections Program in Bangkok. The questionnaire was designed to be administered in Thai via face-to-face interviews, with responses entered in PDAs using GeoAge FAST software. Not all questions provided data used in this study. The questionnaire was developed based on socio-ecological reasoning about gaps in rabies knowledge that potentially translate into failed prevention on the individual level.

Data were collected on demographics; primary bat-associated activity and years of experience; history of rabies vaccination; and type and frequency of bat exposures such as cave entry, direct contact with bats, bites and scratches from bats, and bat consumption. Individuals who reported receiving rabies vaccination were asked to indicate whether it was in direct response to an animal exposure (i.e. PEP) or for pre-exposure immunization (PreP), which is a vaccination series most often administered to people who have a relatively high likelihood of rabies virus exposure due to occupational risks or other factors. Those who reported receiving PreP were asked to describe its administration and only those who indicated receiving a series of injections spaced over multiple days were counted as having PreP.

To assess rabies-related knowledge, participants were asked to rate their understanding of the disease as either “little or none”, “basic”, or “extensive”; explain how humans acquire the disease, and identify animal sources of the disease. Each knowledge question was evaluated independently, and the validity of a participant's self-reported knowledge level was not verified using other responses. Participants were also asked to describe the severity of rabies. Only responses that emphasized death or profound suffering with no suggestion that recovery was likely were considered evidence that the participant recognized rabies as being severe. Awareness of other diseases that humans can get from bats was also elicited.

To assess health-seeking practices following transdermal bat exposures, participants were asked about actions they would take if they were bitten or scratched by a bat. Responses to this open-ended question were compared to a similar question later asked about actions a person should take following a bite from a potentially rabid animal, based on the participant's own understanding of what constitutes a potentially rabid animal. Questions that were specifically asked about bats preceded all questions asked about rabies to minimize reporting bias, and whenever feasible, participants were asked open-ended questions to minimize the interviewer's influence on responses. Participants were also interviewed away from other people. Interviewers were instructed to not ask questions in a leading manner and to allow as much time as necessary for participants to answer.

### Statistical analysis

Survey responses were transferred to a computer, exported into Microsoft Excel, and then imported into SAS version 9.2 for analysis. Data were summarized using descriptive statistics and comparisons by bat-associated activity group were made using Chi-square or Fisher's exact test. Multivariable logistic regression analysis was used to explore factors independently associated with being bitten or scratched by a bat. Variables related to the outcome at p-values≤0.25 were included in the model. Crude and adjusted odds ratios (OR) with 95% confidence intervals (CI) were calculated. Associations were statistically significant at p-values less than 0.05.

## Results

The study was conducted during August 3–18, 2009. A total of 106 people were interviewed. Interviews lasted an average of about 10 minutes. Demographic characteristics, history of rabies vaccination, and primary bat-associated activity of the participants are described in Table 1. All temple workers/residents and game wardens were involved in their activity at the time of interview; 71% of guano miners and 53% of bat hunters reported that their most recent engagement had occurred within the previous 12 months. Of all groups, guano miners had fewer years of schooling, with 89% educated at the primary level or less versus 55% of non-guano miners (p = 0.001). Temple workers/residents were more likely to have greater than 15 years of experience in their activity than other groups (59% vs. 26%, p = 0.001).

**Table 1:** Description of participants by demographics, activity, and rabies vaccination history.

Category	Subcategory	Value^a^
No. (%) male	–	86 (81)
Age in years, median (range)	–	44 (20–75)
No. (%) with < primary level^b^ education	–	68 (64)
No. (%) with >15 years of experience^c^	–	41 (40)
Bat-associated activity, No. (%)	Temple work/residence	41 (39)
	Guano mining	28 (26)
	Game warden	19 (18)
	Bat hunting	18 (17)
Prior rabies vaccination, No. (%)	None	73 (69)
	PreP^d^	8 (8)
	PEP^e^	25 (24)

a Percentages are rounded.

b Six years or less of formal education.

c More than 15 years of experience in bat-associated activity.

d PreP = Pre-exposure prophylaxis.

e PEP = Post-exposure prophylaxis.

Thirty-one percent of participants reported a history of receiving either rabies PreP (7.5%) or PEP (23.5%) within their lifetimes. Of those who had received rabies PEP, 96% reported that they had been vaccinated in response to a dog exposure and 4% for a cat exposure. No participants reported receiving PEP for a bat exposure and none reported receiving both PreP and PEP. There were no statistically significant differences between activity groups with respect to rabies vaccination.

Table 2 describes participant's responses to rabies-related knowledge questions by activity group. A majority of participants (54%) reported having little or no knowledge of rabies. Proportionately more temple workers/residents reported basic or extensive knowledge than non-temple workers/residents (p = 0.03). Self-assessed rabies knowledge appeared to be lowest among guano miners, but not to a statistically significant degree (p = 0.06). Although most (85%) participants seemed to be aware that animal bites cause rabies, significant differences were observed between activity groups. Only 68% of guano miners indicated animal bites as a mechanism of transmission compared to 90% of non-guano miners (p = 0.03). When asked to identify which animals are sources of rabies, only 11 (10%) participants named bats. In contrast, dogs were named by 80 (76%), cats were named by 41 (39%), and other mammals (including rodents and large domestic animals) were named by 24 (23%). Fourteen participants (13%) were unable to name any animals as rabies sources. Differences between activity groups with respect to bat attribution were not statistically significant, and individuals who attributed rabies to dogs were no more likely to also attribute rabies in bats than those who did not attribute rabies to dogs (11% vs 8%, p = 1.0) (not shown in Table 2). When asked whether they were aware of any other diseases (besides rabies) that humans can get from bats, 18 (17%) answered yes; all were temple workers/residents (not shown in Table 2).

**Table 2:** Responses (%) to rabies-related knowledge questions, by bat-associated activity.

Rabies-related knowledge response	Temple workers/residents (n = 41)	Guano miners(n = 28)	Game wardens(n = 19)	Bat hunters (n = 18)	Total(n = 106)
Reported having little or no rabies knowledge	39	77	53	65	54
Indicated animal bites as mechanism of transmission	95	69	89	94	85
Described rabies as severe	83	65	89	71	78
Identified bats as a rabies source	5	7	16	22	10

Table 3 shows how participants responded when asked “What actions would you take if you were bitten or scratched by a bat?” and “If someone has been bitten by an animal potentially infected with rabies what should that person do?” Twenty-eight (26%) participants expressed that they would seek medical care or rabies PEP for a bat bite or scratch, while a significantly higher proportion (95%) advocated these actions if the bite came from a potentially rabid animal (p = 0.0001). The proportion of participants who either said they would do nothing or that they didn't know what they would do if bitten or scratched by a bat was significantly higher than the proportion answering similarly when asked about an exposure to a potentially rabid animal (36% vs. 2%, p = 0.0001). Guano miners were more likely than non-guano miners to give this response for bat exposures (61% vs. 27%, p = 0.003). An incidental finding (not shown in the table) was that previous recipients of PreP or PEP were more likely than non-recipients to advocate a health-seeking behavior for bat bites and scratches but not to a stasticially significant extent (82% vs 56%; p = 0.15).

**Table 3:** Percentage of participants advocating specific actions in response to bat or rabid animal exposure.

Advocated action	For a bat exposure	For exposure to a potentially rabid animal	p-value^a^
Seek medical care/rabies PEP	26	95	<0.0001
Wash wound^b^	43	33	0.09
Nothing/don't know	36	2	<0.0001
Traditional care^c^	0	3	0.24
Other^d^	8	1	0.04

a Comparing the proportion of participants who advocated a given action for a bat exposure with the proportion who advocated the same action for an exposure to a potential rabid animal.

b Includes cleaning the wound with water, soap, and/or a common antiseptic agent (e.g. betadine).

c Includes prayer or visiting a traditional healer.

d Includes bleeding the wound, bandaging, or using topical home remedies (e.g. local herbs).

Table 4 describes bat-related exposures by the number and proportion of participants who reported experiencing it at least once in their lifetimes, along with those who reported experiencing it more than five times a year. A history of transdermal bat exposure (bite or scratch) was reported by 29 (27%) participants.

**Table 4:** Bat-associated exposure histories reported by participants.

Exposure Type	No.(%) ever having exposure	No.(%) having exposure >5×/year
Inside bat cave or roost area	96 (91)	61 (58)
Direct bat contact	63 (69)	29 (27)
Bat consumption	57 (54)	11 (10)
Bat scratch	23 (22)	6 (6)
Bat bite	18 (17)	2 (2)

Table 5 shows factors independently associated with a bat bite or scratch history. Variables considered for inclusion in the multivariable model on the basis of biological plausibility included age, sex, years of experience, education, knowledge self-assessment, frequency in bat caves/roost areas, and bat-associated activity. All except the latter three variables were removed from the final model due to unadjusted p-values>0.25. No two variables were so strongly associated with one another or the outcome as to suggest the presence of colinearity. In the final model, self-assessed rabies knowledge of “little or none” was not significantly predictive of a bat bite or scratch history, although a strong association was observed prior to adjustment for other variables. Individuals who engaged in guano mining, bat hunting, or visiting a bat cave or roost area more than 5 times a year were more likely to report a history of bat bites or scratches.

**Table 5:** Factors independently associated with a bat bite or scratch history based on multivariable analysis.

Category	Subcategory	N(%)	OR^a^ (95% CI)	aOR^b^ (95% CI)
Bat-associated activity	Temple worker/resident	7 (17)	ref	ref
	Guano miner	11 (39)	3.1 (1.0–9.6)	6.7(1.8–25.6)
	Game warden	5 (26)	1.7 (0.5–6.4)	3.7 (0.8–16.8)
	Bat hunter	6 (35)	2.4 (0.7–8.7)	12.0 (2.0–72.0)
Self-assessed rabies knowledge	Little or none	21 (38)	3.2 (1.2–8.0)	2.7 (1.0–7.5)
	Basic and above	5 (26)	ref	ref
Frequency of being in a cave/roost area	≤5×/year	7 (16)	ref	ref
	>5×/year	22 (36)	3.1 (1.2–8.0)	10.6 (2.9–39.7)

a OR (95% CI) = Odds Ratio (95% Confidence Interval).

b aOR = Adjusted Odds Ratio.

## Discussion

In this survey among persons at risk for bat exposure in Thailand, we found that although general awareness of rabies transmission and severity were relatively high, awareness of bat rabies in particular was low, with only 10% of participants identifying bats as a potential source of rabies and 36% failing to say they would take any specific action if bitten or scratched by a bat. Bat exposures conducive to potential lyssavirus transmission were also common in this population and were reported by members of all four activity groups, supporting more than just a theoretical risk for these types of incidents. We found that guano miners reported the highest frequency of transdermal bat exposures, were the least knowledgeable about rabies, and were the least likely to say they would respond to bat exposures in a manner that would ensure rabies prevention. Based on these findings we conclude that of the groups we surveyed, bat rabies has the greatest potential impact on guano miners. The potential risk associated with guano mining is even more stark given that moribund bats (i.e., those most likely to be rabid) normally fall to the floor of caves, where they can readily come in contact with someone collecting bat droppings by hand. The effectiveness of any bat-borne rabies prevention strategy may hinge upon how well it diffuses into communities where guano mining regularly occurs.

Education at the community level is an important strategy in the prevention of human rabies [4]. Although the decreasing incidence of human rabies in Thailand points to the effectiveness of past and present rabies education efforts, our findings demonstrate a need to raise public awareness of the potential risk of rabies associated with bat exposures. Special attention should be placed on communities where bats or bat guano are commonly utilized, and if school-based, programs should include primary level students to ensure that they reach those who do not progress past this level of schooling. In addition to emphasizing the importance of exposure avoidance and countering attitudes that inappropriately lower risk perception towards bats, wound washing and healthcare utilization following bat bites and scratches are practices that should be promoted. Similarly, if the awareness we observed in the public is indicative of awareness in the medical community, outreach to healthcare professionals might also be needed to ensure that patients presenting with bat exposures are treated in accordance with WHO guidelines [4]. Studies aimed at assessing knowledge and practices in the Thai medical community should be explored to ensure that such outreach is well-informed. Education at temples and national parks is also recommended to ensure personnel at these sites know to avoid unnecessary bat contact and respond appropriately to bat-inflicted injury. A a strategy that integrates community outreach with law enforcement should be considered as well.

To date, there have been no reported cases of human rabies cases associated with bats in Thailand. One plausible explanation is that the prevalence of bat lyssaviruses in SE Asia is so low that humans are rarely if ever exposed to these pathogens. It is also possible that the prevailing assumption about dogs as the usual source of rabies leads patients and their family members to overlook relevant bat encounters when recounting animal exposures, resulting in misdiagnosis or misattribution. Additionally, the rate of human rabies vaccination in the population may be high enough to protect many people against bat rabies. In our study, we found that almost a third of all participants reported a history of rabies vaccination, mostly as a result of dog-associated PEP. This suggests that the percentage of individuals in our study population with at least some lyssavirus immunity is relatively high and may help account for why bats have yet to be linked to any human rabies cases in the country. However, immunity levels could change if PEP use becomes more conservative in the future. Currently, funds annually spent on the purchase of human rabies biologics by the Thai government are quite substantial [8], and this financial burden may be difficult to sustain indefinitely.

There are some limitations to our study that should be noted. First, it is unlikely that our relatively small convenience sample is representative of all persons engaged in bat-related activities in Thailand. Our findings may have also been subject to reporting bias, since guano miners and bat hunters may have been less willing than others to answer questions truthfully due to the illegal nature of their work. This potential bias may have led participants to understate their years of experience, which could explain why this variable was not found to be associated with a history of transdermal bat exposures. Estimated participation rates for these two groups were also much lower than the other two groups (participation rates were hard to definitively ascertain because participation was ultimately premised on self-identification). Additionally, we classified individuals based on their self-reported *primary* bat-associated activity; however, a few participants indicated involvement with other activities (e.g,. guano miners that also hunt bats) either presently or in the past. Having such a history was not accounted for this study, although it potentially could be associated with an increased lifetime risk of transdermal bat exposures. The desired sample size of 200 persons was somewhat arbitrarily determined given the lack of reliable estimates for the study population size. Failure to meet this number was largely due to the difficulty in finding willing participants who engaged in bat hunting and guano mining, and the limited availability of personnel and funds that could be used to extend the study period. As a consequence of our small sample size and low statistical power, truly significant associations may have gone undetected in this study. However, by recruiting from several provinces, we minimized the influence that geography might have imparted on the associations we observed. Another limitation is that the validity and reliability of the questionnaire may have been suboptimal because the survey instrument was not subject to very rigorous in-field testing.

Our findings have relevance to zoonotic diseases other than rabies. SE Asian bats have been linked to the encephalitis-causing Nipah virus and Hendra virus [23],[24],[25], and the corona virus associated with severe acute respiratory syndrome (SARS) [26], [27]. Less novel diseases associated with bats also include histoplasmosis, an invasive fungal respiratory disease linked to bat guano exposure [28]. Additionally, evidence suggests that bat ectoparasites may transmit pathogens such as bartonella and rickettsia [29],[30]. In this study, we found that exposures that could potentially facilitate transmission of these diseases appear to occur relatively frequently, with 36% of surveyed participants reporting that they experience direct contact with bats at least twice a year. Bat consumption—an activity that in and of itself may be low risk (assuming the bat is well cooked) but could be associated with increased disease risk through contact with bat carcasses—was reported by more than half the participants. Exposure to toxic or infectious aerosols is another potential hazard for this population as well, since almost all participants reported regularly being in bat caves and roosting areas. More epidemiological studies are needed to better assess the risks associated with bat-related exposures, particularly in regions of the world where outbreaks of severe zoonoses have occurred and questions remain regarding animal reservoirs for such diseases.
